# Quantification of Microstructural Changes in the Dermis of Elderly Women Using Morphometric Indices of the Skin Surface

**DOI:** 10.3390/ma15228258

**Published:** 2022-11-21

**Authors:** Manon Bachy, Catherine Bosser, Benoît Villain, Jean-Charles Aurégan

**Affiliations:** 1 B3OA, UMR7052, Paris University, 75010 Paris, France; 2 Department of Paediatric Orthopaedic Surgery, Armand Trousseau Hospital, Assistance Publique–Hôpitaux de Paris, Sorbonne University, 75012 Paris, France; 3 HealthDataSciences, 45 Chemin du Barthélémy, 69260 Charbonnières les Bains, France; 4 Department of Orthopaedic, Trauma and Reconstructive Surgery, Antoine Béclère Hospital, Assistance Publique–Hôpitaux de Paris, Paris Saclay University, 92140 Clamart, France; 5 CIAMS, Paris-Saclay University, 91405 Orsay, France; 6 CIAMS, Orléans University, 45067 Orléans, France

**Keywords:** skin, ageing, microstructure, collagen, osteoporosis, skin replica

## Abstract

Objective: The main objective of this study was the development of a non-invasive mathematical marker of the skin surface, the characteristic length, to predict the microstructure of the dermis. This marker, at the individual level, is intended to provide the biological age of the patient in the context of personalised medicine for the elderly. Study design: To validate this hypothesis, a clinical study was conducted on 22 women over 60 years old from a population of osteoporotic subjects who sustained a femoral neck fracture: a morphological analysis of the skin surface was performed on the patient’s forearm and quantitatively compared with microarchitectural parameters of the dermis. Major results: The Elastin-to-Collagen ratio measured on dermis samples ranged between 0.007 and 0.084, with a mean of 0.035 ± 0.02. The surface characteristic length ranged between 0.90 and 2.621, with a mean of 0.64 ± 0.51. A very strong correlation was found between this characteristic length and the Elastin-to-Collagen ratio (r = 0.92). Conclusions: This study proposes an original diagnostic tool based on morphometric indices of the skin surface and shows a direct quantitative relationship with the dermis microarchitecture and its collagen and elastin content. The proposed method allows reliable and easy access to the intrinsic ageing of the dermis, which would be a strong biomarker in a personalised collagen treatment approach.

## 1. Introduction

Ageing is a common process taking place in every subject of a population, leading to increasingly complex patient care. However, the effect of ageing at the individual level is highly variable since chronological age does not automatically reflect biological age, which varies greatly from one individual to another, depending on intrinsic factors such as genetic inheritance and chronic pathologies (diabetes, asthma, hypertension), as well as extrinsic factors related to lifestyle habits (smoking, alcohol, exercise) [1]. The search for a better quality of care requires taking into account the patient’s specific clinical particularities. In this context, it is necessary to have specific markers of ageing for each individual in order to estimate the differences between chronological age and biological age. With increasing life expectancy, the senior population is obviously the one where the difference between chronological and biological age is the greatest and the most sensitive, but also the one where the need for predictive tools for prevention strategies is the most crucial.

Due to its accessibility and its role as a barrier between the exterior and interior of the human body, skin tissue is a very good candidate for assessing this ageing difference. It is in direct contact with the external environment and fulfils various physiological functions, both protective (ultraviolet rays, infections…) and regulatory (temperature, hydration…). Furthermore, while some parts of the body, such as the face, are highly affected by environmental factors, other parts, such as the forearm and the gluteal region, are mainly the results of the intrinsic ageing of each individual [2]. The skin is a complex structure composed of several distinct layers; in general, the three main layers considered are the epidermis, dermis, and hypodermis. The epidermis, the outermost layer, can itself be subdivided into several layers, including the stratum corneum, which is in direct contact with external environmental conditions. Its properties are particularly important for the friction mechanism, and its alteration affects the distribution of deformation in the underlying layers [3]. Because of its relative size and high collagen content, the dermis is generally considered the main supporting layer of the skin. The two main components of the cutaneous extracellular matrix of the dermis are the structural proteins: collagen (70–80%) for strength and elastin (2–4%) for skin elasticity [3,4]. They are responsible for the network of wrinkles and fine lines that divide the surface of the skin into irregular geometric shapes, which form the microrelief or the particular texture of the skin [5].

The structural evolution of the dermis and skin surface changes inexorably throughout life, related to intrinsic ageing or sun exposure, are well documented in the literature [2,3,4,5,6,7,8,9,10]. In all these studies, the main determinant is the chronological age of the patients, which ranges from 10 to 90 years. Several methods, such as two-photon microscopy, multiphoton microscopy, and multiphoton laser scanning tomography, have been used to explore skin microstructure and to relate it to ageing [2,3,4,5,6,7,8,9,10].

The study of the skin surface, often based on roughness parameters, is usually performed on a skin replica, which is a simple, proven procedure and a convenient way to study the skin independently of its support [11,12]. The transcription of the skin surface can be subjected to various physical or mechanical manipulations that the skin would not have endured. Analysis of the dermis and, in particular, elastin-collagen ratios are usually measured by multiphoton microscopy (MPM), which is an efficient high-resolution technique used successfully in dermatology, including the ageing of the skin. This technique provides the possibility to differentiate the autofluorescence (AF) signal mainly emitted by elastin fibres and the second harmonic generation (SHG) signal mainly induced in ordered structures such as collagen [2,6,7,8,10,13,14,15,16]. Results show that in young people, the collagen fibres of the dermis are entangled and keep the skin taut, as the shapes of the microstructure are complicated. On the other hand, with age, the skin sags, the furrows deepen, and the microstructure changes and disappears [3,4]. In addition, it has been known for a long time that the evolution with the age of the subjects between the characteristics of the dermis and the surface of the skin in women is different from that of men [17]. Similarly, often in these studies, a great variability is observed from one individual to another within the same age group, and this variability increases notably after 60 years of age, especially in women [16,18,19]. In this context, although trends in skin surface degradation and dermal microstructure are concomitant, inter-individual variability is a challenging factor that should be included to obtain a reliable and patient-specific approach based on chronological age to help medical decisions providing a specific therapeutic approach based on patient characteristics.

Thus, the main objective of this study was the development of a non-invasive mathematical marker of the skin surface to predict the microstructure of the dermis. This marker, which is lacking at the individual level, is intended to provide the biological age of the patient in the context of personalised medicine in the elderly.

We relied on the development of information technologies which have brought to light a new generation of microscopic imaging and computer image analysis allowing the study of morphometric parameters of the skin surface in a quantitative way. Automatic textural analysis of the skin surface has been implemented to access the architectural parameters of the skin. This approach is supported by the hypothesis that microstructural changes in the dermis related to elastin and collagen content can be assessed by morphometric indices of the skin surface.

To validate this hypothesis, a clinical study was conducted on 22 women over 60 years of age from a population of osteoporotic subjects who sustained a femoral neck fracture: a replica of the skin surface was made on the patient’s forearm, and skin samples were taken for microstructural analysis of the dermis in an area not exposed to the sun.

## 2. Methods

### 2.1. Population

In the present experimental study, 22 women over 60 years of age with a femoral neck fracture were included. All patients were managed consecutively in the orthopaedic surgery department of the University Hospital Antoine Béclère, France. Patients with a medical history, treatments or habits competing with metabolism (term corticosteroids, smoking, alcohol consumption > 3 units/day) or a bone or skin pathology were excluded [20]. The mean age was 82.6 years (±9.2 years, range 61–96). The mean height was 160.8 cm (±6.0 cm, range 146–171) and the mean weight was 60.8 kg (±9.1 kg, range 40–75). The mean BMI was 23.5 kg/m^2^ (±3.4 kg/m^2^, range 17.8–30). For each patient, a 5 × 5 mm^2^ skin biopsy was taken in the surgical area in the gluteal region and stored at −20 °C until further processing. At the same time, a standardised oriented silicone skin replica (SILFLO, silicone polymer and catalyst Monaderm, Rue des Violettes, Monaco) was made at the anterior aspect of the right forearm, 5 cm distal to the elbow, toward the Flexor Carpi Radialis tendon relief. The study was conducted in accordance with the Declaration of Helsinki and was approved by the ethics committee of Paris Sud (reference no: PP-14-018). All the patients gave their written consent before participating in the study.

### 2.2. Microstructural Dermis Evaluation: The Elastin-to-Collagen Ratio

For all patients, the upper dermis of the collected skin biopsy was imaged by two-photon confocal imaging (A1RMP PLUS^®^, Nikon, Tokyo, Japan) using an excitation wavelength of 900 nm. Second harmonic generated (SHG) light from collagen and autofluorescent (AF) light from elastin were collected on two channels with specific band-pass filters of 400–490 and 500–550 nm, respectively. A 25×, 1.1-NA water immersion objective (CFI Apo LWD 25XW, Nikon) was used. The image field of view was 512 × 512 µm^2^ with a resolution of 0.5 µm/pixel. Stacks of 2D images were recorded with a time span of 4 s per image, every 1 µm in depth.

For each sample, obtained pictures for elastin and collagen were treated with a customised Python algorithm (v3.7, Python Software Foundation, Wilmington, DE, USA), based on the k-Means clustering algorithm that is a non-parametric method that will separate the observations into k groups, based on Euclidian distance [21]. In the first channel, related to the collagen signal, k-Means clustering was used to determine the grey level threshold to separate the collagen signal from the background signal (k = 2). In the second channel, related to the elastin signal, k-Means clustering was used to determine the grey level threshold to separate the elastin signal from the collagen, also partially visible and background signals (k = 3). The centroid values of the clusters labelled collagen and elastin, respectively, were chosen as the collagen and elastin grey level threshold. Based on these automatic thresholds, the Elastin-to-Collagen ratio (*R_EC_*) was then calculated for each sample as the number of elastin pixels over the number of collagen pixels in the entire stack of the image.

### 2.3. Skin Surface Analysis

The skin surface from the replica was analysed in two modalities: roughness measurement and morphometric analysis.

The roughness index, *Ra*, was measured both in the longitudinal direction, parallel to the axis of the forearm, and in the transversal direction, along a line length of 10 mm in the centre of the replica with points spaced at 1 µm according to the following equation:

(1) $$Ra=\frac{1}{l}\int_{0}^{l}\left|z\left(x\right)\right|dx$$

where *z*(*x*) is the distance in the vertical direction between the replica surface and the mean line of the profile, within the evaluation length *l*. The vertical distance profile was acquired using a chromatic confocal optical pen with a measurement range of 1.4 mm (CL3-MG70, Stil, Aix-en-Provence, France) associated with a custom translation stage and was processed using Python routines that remove baseline offsets, centre profile around its mean and calculate the roughness *Ra*. The *Ra* measured value of a glass slide is 92 nm, giving the error of the whole acquisition and processing chain. In the following, the roughness index value, *Ra*, is defined as the average of the roughness index of the longitudinal and transversal profiles.

**Figure 1:**
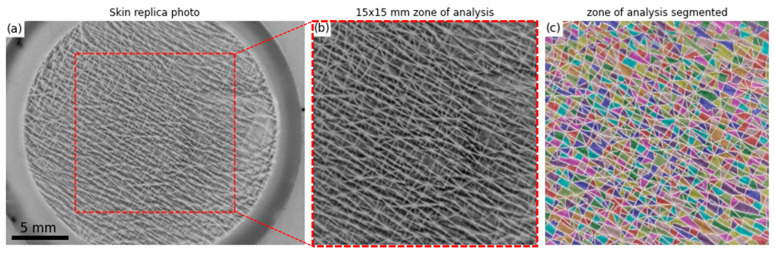
(**a**) HQ skin replica picture (**b**) Crop of a 15 × 15 mm zone of analysis in the central part of the photo (**c**) Results of Marker-Controlled Watershed segmentation.

Morphometric analysis was performed using a radiant circular led light and a 12 million pixels camera (Raspberry Pi HQ, Raspberry Pi Foundation, Cambridge, UK), allowing the acquisition of the skin patch image (Figure 1a). A 15 × 15 mm analysis area in the central part of the patch photo was selected and defined as a grayscale (Figure 1b). As a cutaneous replica, the lowest points correspond to the basins, which are the skin polygons (black = 0) and the highest points to the wrinkle (white = 255). Image segmentation was processed with a Marker-Controlled Watershed algorithm that treats pixel values as local topography and floods basins from markers. The success of this transformation depends on the choice of markers. Mathematical morphology, in a customised Python routine, was used to compute the extended regional minima after an H-minima transform [22,23] (Figure 1c).

Then, several morphometric skin features such as the number of polygons per mm^2^ (*NP*) and, for each of them, their area (*A_i_*), perimeter (*P_i_*), orientation, minor (*m_i_*), and major (*M_i_*), aspect ratio ($R_{Asp,i}=\frac{M_{i}}{m_{i}}$) were extracted. A characteristic length scale (*Lc*), averaging the sum of the equivalent diameter ($\sqrt{A_{i}}$) and equivalent side ($\frac{P_{i}}{4}$) of the polygons, was also calculated.

### 2.4. Statistical Analysis

Continuous data are presented as mean, standard deviation and range of values. All statistical tests were performed using R (R Foundation for Statistical Computing, Vienna, Austria). Mann–Whitney U-tests were performed to assess statistical differences between groups, with a significance level of 0.05. Pearson correlation analyses were used to evaluate the correlations between skin replica data and skin quality (*R_EC_*).

## 3. Results

**Figure 2:**
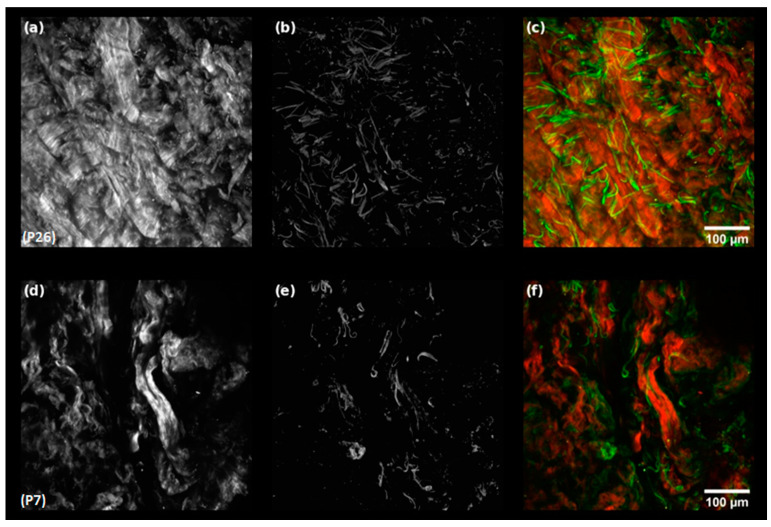
Representative multiphoton images of the dermis of cutaneous biopsies. Sample with lowest *R_EC_* value (P26): (**a**) collagen fibres, (**b**) elastin fibres, and (**c**) false colour overlay image (red: collagen fibres; green: elastin fibres); Sample with highest *R_EC_* value (P7): (**d**) collagen fibres, (**e**) elastin fibres, and (**f**) false colour overlay image (red: collagen fibres; green: elastin fibres).

The Elastin-to-Collagen ratio ranged between 0.007 and 0.084, with a mean of 0.035 ± 0.02. Figure 2 shows SHG, AF and false-colour overlay images of the minimum (Figure 2a–c) and maximum (Figure 2d–e) *R_EC_* values samples. Collagen and elastin fibres could easily be identified in the SHG and AF images, respectively. In the low *R_EC_* sample, a dense collagen network with thick intertwined bundles is visible (Figure 2a) and elastin fibres appeared as distinct thin bundles (Figure 2b). The false-colour overlay image, with red representing collagen fibres and green representing elastin fibres, showed that the two types of fibres spatially intertwine with each other (Figure 2c). In the highest *R_EC_* sample, the collagen network structure was altered (Figure 2d), and fibres were unravelled while elastin fibres (Figure 2e) were fragmented.

**Figure 3:**
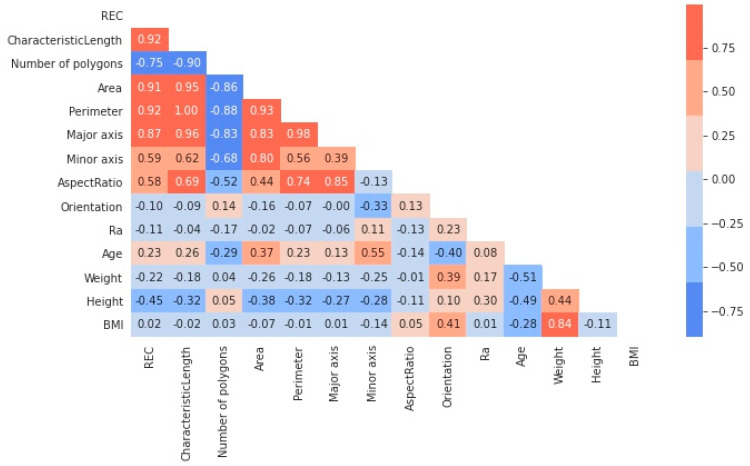
Heatmap of correlation coefficients (r) between all data (skin Elastin-to-Collagen Ratio, skin replica segmentation, roughness, and demographic). The stronger red and blue colours would then indicate strong positive and negative correlations, respectively.

The results of skin surface roughness measurement and morphometric analysis are summarised in Table 1, and a heatmap of absolute correlation coefficients (*r*) between all data: demographic, Elastin-to-Collagen ratio, morphometric analysis and roughness are given in Figure 3.

In the present study, considering a population of women over 60 years of age, the best correlation coefficients are reported between the Elastin-to-Collagen ratio and the morphometric parameters, whereas the patient’s age and roughness show a lower correlation. Therefore, samples were grouped according to the characteristic length values to perform the statistical analysis: samples with a characteristic length below the median value were assigned to a « low_Lc » group; samples with a characteristic length above the median value into a « high_Lc » group. The detailed results of the statistical analysis between the two groups are summarised in Table 2. The Elastin-to-Collagen ratio was significantly higher in the « high_Lc » group, whereas no significant differences were found between the two groups with respect to patient data: age, height, weight, or BMI.

**Figure 4:**
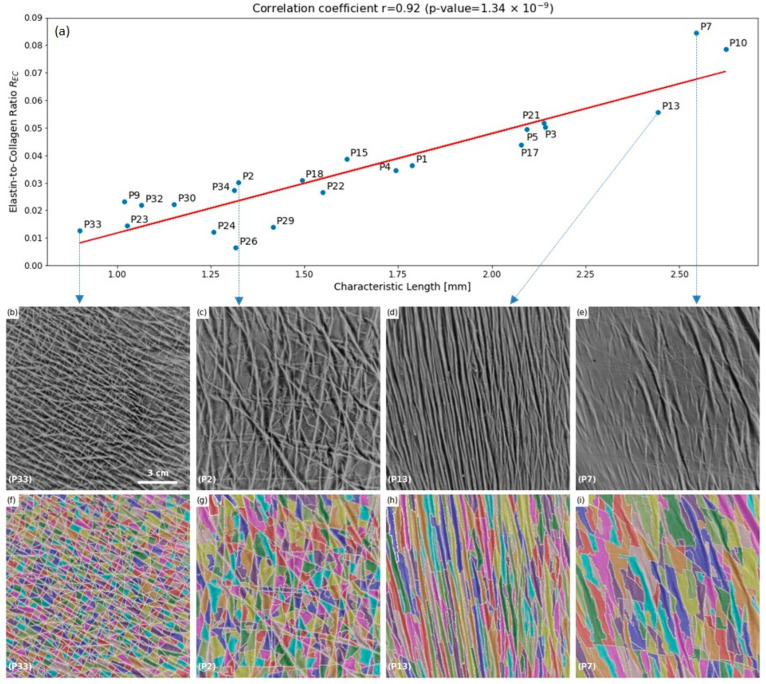
(**a**) Very strong correlation was found between Characteristic Length and the Elastin-to-Collagen ratio. Optical classification on raw (**b**–**e**) and segmented (**f**–**i**) images from lower SL/*R_EC_* at left to higher SL/*R_EC_* at right. (**b**,**f**) patient P33 (67 years old) showed a network of lines distributed in all directions in an isotropic and very dense manner; (**c**,**g**) patient P2 (92 years old) showed wider and more distant lines leading to a decrease of density with a slight predominance of vertical lines; (**d**,**h**) patient P13 (87 years old) showed only a set of parallel lines, all directed in a privileged direction close to the arm axis; (**e**,**i**) patient P7 (85 years old)showed almost no more lines.

From a visual point of view, the morphological analysis reveals four main types of patterns that can be qualitatively related to the skin surface change. Figure 4 illustrates these four main types obtained from the optical images and their segmentation at different points on the curve between characteristic length and Elastin-to-Collagen ratio. This qualitative classification into four groups clearly reflects changes in the microstructure of the upper dermis, ranging from an isotropic distribution of tension lines for patients with the lowest characteristic length to a total loss of tension lines for patients with the highest characteristic length values.

## 4. Discussion

Skin ageing leads to structural disorders that limit the functional capacities of the skin and attenuate its protective effect, which is associated with immune dysfunction. These age-related alterations of the skin, compromising the structural integrity and barrier function, have the potential to cause health issues. As a consequence, skin infections are more frequently observed in elderly patients, as the development of chronic wounds provides a gateway for infectious agents [24,25]. In the context of increasing life expectancy, the search for mechanisms to restore skin function is very active, and in particular, drugs that can induce the synthesis of type I collagen, such as the fibroblast growth factor (FGF), are of great interest [26]. At the same time, it is essential to have markers of skin ageing in this specific elderly population to identify and classify the individuals most at risk in order to prescribe appropriate care.

Indeed, degradation of structural [6,7,8,10,13,14,15,16,27,28,29], mechanical [3,30,31,32], textural [5,9], functional and physiological [4,33,34] aspects of the skin are now well identified between a young and old population, but a specific marker of skin ageing in the older population is lacking. In the present study, the multiscale characterisation approach of dermis and skin surface microstructure implemented on a population of older women shows a direct relationship between the evolution of the structural components of the dermis, elastin and collagen, and morphological changes of the skin surface, in the context of intrinsic ageing. To the authors’ knowledge, this is the first clinical study based on reliable and elegant indices of skin surface morphology to establish the quality of the dermis in this elderly population. The index obtained in this study is totally uncorrelated with the chronological age of the patient, thus revealing the specificities of each subject, whether genetic or environmental, mainly related to the behavioural factors of each individual.

In the present study, the Elastin-to-Collagen Ratio was determined using two-photon confocal imaging and a k-Means clustering algorithm allowing automatic quantification. Several independent studies have investigated quantitative analysis of collagen and elastin in the skin to determine the effect of ageing, photoaging, disease, gender, and body area. Some studies scored the evolution of fibre orientation and anisotropy based on Multiphoton Microscopy (MPM) [10,13,16] or viscoelastic properties of the skin [31]. Koehler et al. introduced the MPM-based Dermis Morphology Score (MDMS) based on the identification of morphological patterns of collagen and elastin fibres [14]. Lin et al. proposed in 2005 the SHG-to-AF ageing index of the dermis (SAAID) [18], which is based on signal intensities in AF and SHG channels and defined by SAAID = (I_SHG_ – I_AF_) /(I_SHG_ + I_AF_). SAAID was used in numerous studies ex vivo or in vivo [2,6,13,15]. Puschmann et al. suggested the use of the Elastin-to-Collagen ratio (ELCOR) as the ratio of the area fraction of the full image occupied by elastin fibres to the area fraction of the full image occupied by collagen fibres. However, ELCOR and SAAID suffer from certain limitations: (i) SAAID used only small regions of interest (ROI) of the entire AF and SHG images that are subjectively selected, so the collagen/elastin fluctuations have to be averaged on a large scale to get physiological information; (ii) SAAID is difficult to compare as the values depend of intensities, that is highly dependent of the experimental parameters (power of laser, gains and offset of each channel); (iii) ELCOR and SAAID assumed that SHG and AF channels contain only pure signals of collagen and elastin respectively; (iv) SAAID, and ELCOR analysed only a 2D image [15]. It is surprising that the reported values of ELCOR are so high, close to 0.5, in light of the composition of the dermis: collagen 70–80% and elastin 2–4% of the dry weight of the skin [3,4]. The ratios obtained in the present study ranging from 0.007 to 0.084, are consistent with the values derived from the composition, which should be between 0.025 and 0.06. The Elastin-to-Collagen ratio was determined on an entire 3D image. The imperfections of the acquisition signal related to the presence of the collagen signal in the AF channel were corrected. The background signal and collagen traces in the AF signal were removed using the k-Means clustering method (k = 3) so that the elastin signal could be properly separated.

To date, all methods agreed to reveal that the relative collagen content decreases with intrinsic ageing. This can be attributed to a decrease in collagen synthesis; or/and to an accumulation of partially degraded elastin fibres [4,14]. Thus SAAID was found to decrease with age [2] as ELCOR significantly increased with age [15]. Collagen and elastin fibre analyses conducted by Wang et al. also confirmed that elastin fibre density increased with age, whereas collagen fibre density decreased with age [16]. However, the results of the present study showed that, in an elderly population, the main determinant of the Elastin-to-Collagen ratio was not related to the age of the subject but varied more individually.

At the skin surface, the morphological organisation in skin texture depends on the anisotropy of the tension lines, termed ‘Langer’s lines’, discovered by Dupuytern and Langer [32]. The cutaneous relief is mainly composed of lines (furrows, wrinkles) that are categorised as primary and secondary lines, according to their width and depth, and form a network-like structure on the skin surface [11]. Depending on the area, the morphology forms a network of various polygons: rectangles, squares, rhomboids, trapezoids, or triangles [9,11].

The morphology of the skin surface is primarily attributed to the dermal matrix, consisting predominantly of collagen and elastin that form an entangled network of fibres. In young adults, collagen fibres are randomly oriented. They form a mesh of fine fibres and small bundles in the papillary dermis and large, weakly intertwined wavy bundles in the reticular dermis. Ageing is associated with a decrease in collagen content, a straightening of collagen fibres forming looser bundles, and an increased crosslinking that restricts slippage inside the network of collagen fibres. From the age of 30, the elastic fibres of the upper dermis tend to disappear and become thinner and fractionated. Moreover, ageing leads to a thinning of the epidermis. All these changes contribute to the formation of wrinkles and the flattening of the skin surface while keeping a certain morphological organisation [3,4,31].

Surface topography is widely used in dermatological practice and research. Roughness parameters were defined as evaluation criteria to study intrinsic and extrinsic skin ageing [5,9,35,36] or to evaluate the effectiveness of preventive (or anti-ageing) treatments [37]. These studies showed that roughness increases from young to aged adults. For instance, Li et al. quantified the evolution of average roughness (*Ra*) from 16.9 ± 3.4 µm for 20–29 years to 28.5 ± 6.8 µm for 60–74 years [5]. However, if the topography seems to be a discriminating parameter for this kind of study, it is difficult to apply in elderly subjects who can present very deep wrinkles or very loose skin almost without relief. Indeed, in the present study, the average roughness varied from 15.2 to 107.5 µm between individuals. In addition, for the lowest roughnesses, which were of the same order of magnitude as those of young adults, the result of ageing was mainly visible in the aspects of the morphological organisation. Within an older population, roughness is not particularly relevant for monitoring the effects of alterations in the microstructure of the dermis on the skin surface.

Other studies focused on surface patterns such as the number of polygons, mean area of polygons, and length of wrinkle [9,19,22] according to age. Vörös et al. found a decrease in the number of polygons per mm^2^ from 11.1 in children, 5.8 in young adults and 3.7 in older adults [9,19], which is consistent with the values found in this study.

Finally, the parameter proposed here, the specific length, allows us to consider both the surface and the perimeter of the polygons, which depend on the quality of the elastin and collagen network. A low characteristic length corresponded to a network of lines distributed in all directions in an isotropic manner, associated with a low Elastin-to-Collagen ratio, and a high characteristic length corresponded to a loosening of these networks, associated with a high Elastin-to-Collagen ratio. Rather than chronological age, characteristic length could be used as an indicator of biological age that can be applied as an individualised criterion supporting decisions on skin treatment strategies [38].

## 5. Study Limitations

The present study has several limitations. The study population is a very specific population of older women with a first femoral neck fracture. Apart from ethical considerations, this choice was guided by the expected large inter-individual diversity, which is often found in studies carried out on osteoporosis. However, the results cannot be directly transposed to the male gender. The analysis of the dermis is only based on the Elastin-to-Collagen ratio. An in-depth analysis at the cellular level using immunohistochemistry could provide a better understanding of the pathobiological aspects of skin ageing, as the study of other important parameters such as dermal microvascular density. Finally, additional comparisons seem mandatory to extend the use of morphometric indices of the skin surface for the estimation of other anatomical areas of fragility.

## 6. Conclusions

In conclusion, the present study is the first step towards a direct link between skin morphological indices and dermis quality. Because of its easy implementation and its speed of execution, the morphometric analysis of the skin surface can lead to the development of more elaborate digital solutions based on artificial intelligence and classification algorithms, allowing a useful tool for diagnosis and/or prognosis, which could be used instantaneously during the medical consultation. Moreover, collagen is the most abundant protein in the human body and type I collagen accounts for 90% of the matrix of bone tissue, 85% of the matrix of dentin, 90% of the dermis, and a large part of the abdominal wall and tendons. Based on the hypothesis posed as early as 2005 by Shuster et al. regarding the theory of cross-ageing between all living tissues, according to which quantitative and qualitative alterations of collagen related to intrinsic ageing would occur at the same rate in internal and cutaneous tissues [39,40], characteristic length could be a marker of ageing that completes the markers of quality of other internal dense connective tissues. In conclusion, this study proposed an original diagnostic tool based on morphometric indices of the skin surface to have insights into collagen and elastin content in the dermis.

## Figures and Tables

**Table 1:** Results of skin replica surface analysis: average roughness and watershed segmentation.

Average Roughness [µm]	Number of Polygons/mm^2^ [-]	Mean Area of Polygons [mm^2^]	Mean Perimeter of Polygons [mm]	Characteristic Length [mm]	Aspect Ratio [-]
38.4 ± 20.2 (15.2–107.5)	2.41 ± 1.27 (0.75–5.67)	0.54 ± 0.30 (0.17–1.31)	4.04 ± 1.45 (2.03–6.67)	1.64 ± 0.51 (0.90–2.62)	3.12 ± 1.28 (2.00–7.13)

**Table 2:** Statistical results of experimental data (Elastin-to-Collagen ratio, watershed segmentation results and average roughness) and patient data (age, weight, height, BMI) for samples assigned to low or high characteristic length groups; N.S.D: no significative difference.

		Low_Lc	High_Lc	*p*-Value
*R_EC_*	[-]	0.020 ± 0.008	0.050 ± 0.018	1.98 × 10^−5^
Characteristic Length Lc	[mm]	1.21 ± 0.19	2.07 ± 0.37	2.84 × 10^−6^
Number of polygons/mm^2^	[-]	3.39 ± 1.09	1.42 ± 0.47	1.13 × 10^−5^
Area	[mm^2^]	0.310 ± 0.093	0.763 ± 0.267	1.13 × 10^−5^
Perimeter	[mm]	2.80 ± 0.46	5.25 ± 1.06	2.84 × 10^−6^
Major axis	[mm]	0.914 ± 0.154	1.858 ± 0.419	2.84 × 10^−6^
Minor axis	[mm]	0.424 ± 0.060	0.496 ± 0.112	N.S.D
Aspect Ratio	[-]	2.29 ± 0.28	3.96 ± 1.41	2.84 × 10^−6^
*Ra*	[µm]	40.8 ± 24.1	36.0 ± 17.4	N.S.D
Age	[years]	82.5 ± 8.0	81.3 ± 11.2	N.S.D
Weight	[kg]	60.1 ± 8.6	59.1 ± 9.8	N.S.D
Height	[cm]	161.1 ± 5.7	160.7 ± 7.6	N.S.D
BMI	[kg/m^2^]	23.1 ± 3.1	22.9 ± 3.5	N.S.D
